# Case report: Overlapping syndrome mimicking infectious meningoencephalitis in a patient with coexistent MOG, NMDAR, mGluR5 antibody positivity

**DOI:** 10.3389/fimmu.2022.919125

**Published:** 2022-08-05

**Authors:** Jie Fu, Lilei Peng, Yang Yang, Yang Xie, Zuoxiao Li, Benbing Rong

**Affiliations:** ^1^ Department of Neurology, The Affiliated Hospital of Southwest Medical University, Luzhou, China; ^2^ Department of Neurosurgery, The Affiliated Hospital of Southwest Medical University, Luzhou, China

**Keywords:** overlapping syndrome, meningoencephalitis, myelin oligodendrocyte glycoprotein, metabotropic glutamate receptor 5, N-methyl-D-aspartate receptor

## Abstract

A 38-year-old Chinese Han man presented with fever, headache and difficulty in language expression. The initial cerebrospinal fluid (CSF) analysis revealed lymphocytic-predominant pleocytosis with a normal glucose level, and magnetic resonance imaging (MRI) showed extensive cortical edema in left cerebral hemisphere. He received the antiviral treatment. However, one week later, he developed psychomotor agitation and seizures. Lumbar puncture was performed again and further testing for autoantibodies was conducted in both the CSF and serum. His CSF was positive for anti-myelin oligodendrocyte glycoprotein (MOG), anti-N-methyl-D-aspartate receptor (NMDAR) and anti-metabotropic glutamate receptor 5 (mGluR5) antibodies. He was diagnosed with overlapping syndrome of MOG antibody-related cerebral cortical encephalitis and anti-NMDAR, anti-mGluR5 autoimmune encephalitis. He received intravenous methylprednisolone and immunoglobulin, followed by oral prednisone and mycophenolate mofetil. His psychomotor agitation and seizures were relieved, and he gradually recovered his language expression ability. We reported for the first time a case that was positive for coexistent MOG, NMDAR, mGluR5 antibodies, which was initially misdiagnosed as infectious meningoencephalitis. This case widens the clinical spectrum of the overlapping syndrome recently reported.

## Introduction

Myelin oligodendrocyte glycoprotein antibody-associated disease (MOGAD) is an inflammatory demyelinating disease of the central nervous system (CNS), and typically presents as optic neuritis, transverse myelitis, brainstem demyelination and acute disseminated encephalomyelitis (1). Recently, MOG antibody-related cerebral cortical encephalitis, a rare clinical phenotype of MOGAD, has been reported (2, 3). Of note, atypical clinical manifestations for demyelinating diseases including fever, seizures, headaches, and cerebral cortical symptoms such as aphasia, mental disorder, and memory impairment, are common in MOG cortical encephalitis (1). In addition, MOG cortical encephalitis has been reported to be accompanied by coexisting anti-N-methyl-D-aspartate receptor (NMDAR) encephalitis (4). The NMDAR and MOG are coexpressed on the oligodendrocytic processes, which may explain the coexistence of these antibodies (5). Metabotropic glutamate receptor 5 (mGluR5) belonging to a family of G protein-coupled receptors is found on post-synaptic terminals of neurons and microglia (6). To the best of our knowledge, there are no case reports of coexistent MOG, NMDAR and mGluR5 antibodies in literature. Herein, we describe a rare case of overlapping syndrome with the coexistence of all three antibodies, manifesting as clinical meningoencephalitis, which is a first of its kind. Our study enriches the current literature on overlapping autoimmune syndromes.

## Clinical case

**Figure 1:**
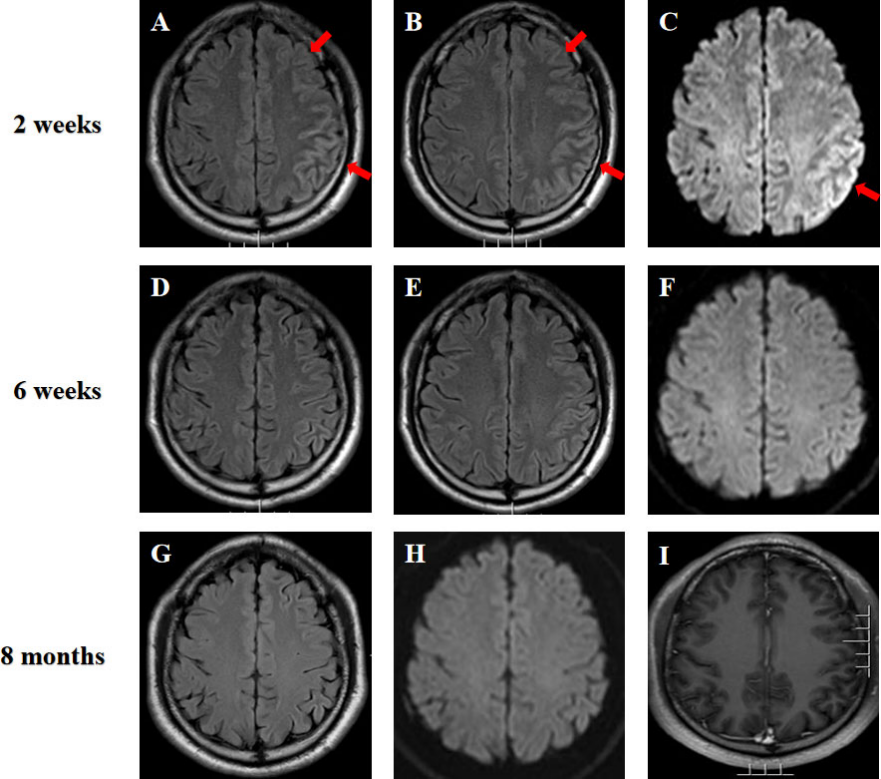
**(A–C)** Brain MRI of the patient at two weeks after the symptom onset showed hyperintensity of the extensive cortex in left cerebral hemisphere on fluid attenuated inversion recovery (FLAIR) imaging and diffusion weighted imaging (DWI) (arrows). **(D–F)** MRI was repeated at six weeks and showed a significant improvement of the imaging abnormality. **(G–I)** MRI performed at almost eight months after initial symptom onset depicted no signal abnormalities on FLAIR, DWI and T1-weighted gadolinium-enhanced sequences.

A 38-year-old right-handed Chinese Han man presented with complaints of difficulty in language expression with no past medical history and no family history of neurological disease. He reported a 12-day history of intermittent fever and headache after catching a cold. On admission, a neurological examination revealed unremarkable except for motor aphasia and neck stiffness. His mini mental score examination (MMSE) was 28/30 (lost 1 point in recall, and lost 1 point in retell). Brain magnetic resonance imaging (MRI) showed extensive cortical edema in left cerebral hemisphere (**Figures 1A–C**). Electroencephalogram (EEG) showed diffuse sharp-slow wave activity in left frontal and temporal lobes (**Supplementary Figure 1**). Complete blood cell count revealed leukocytosis (11.21 * 10^9^/L). Routine biochemical tests, antinuclear antibody and thyroid function testing were normal. Human immunodeficiency virus and rapid plasma reagin were negative. Workup for malignancies (peripheral blood tumor marker, chest and abdominal CT scans) was negative. Cerebrospinal fluid (CSF) analysis indicated an elevated opening pressure (380 mm H_2_O), pleocytosis (white blood cell count 396 * 10^6^/L; 60% lymphocytes), an elevated protein level (1.417 g/L) and a normal glucose concentration. CSF Gram staining, ink staining as well as bacterial and fungal cultures were negative. Testing for infectious encephalitis was unremarkable (CSF PCR for H. influenzae, N. meningitidis, S. pneumoniae, M. tuberculosis, Cytomegalovirus, Enterovirus, Herpes simplex 1 and 2, Varicella zoster, Japanese Encephalitis virus, Epstein-Barr virus and Cryptococcus neoformans). He was empirically treated for presumptive viral meningoencephalitis with intravenous acyclovir, and mannitol was used for the reduction of intracranial pressure.

**Figure 2:**
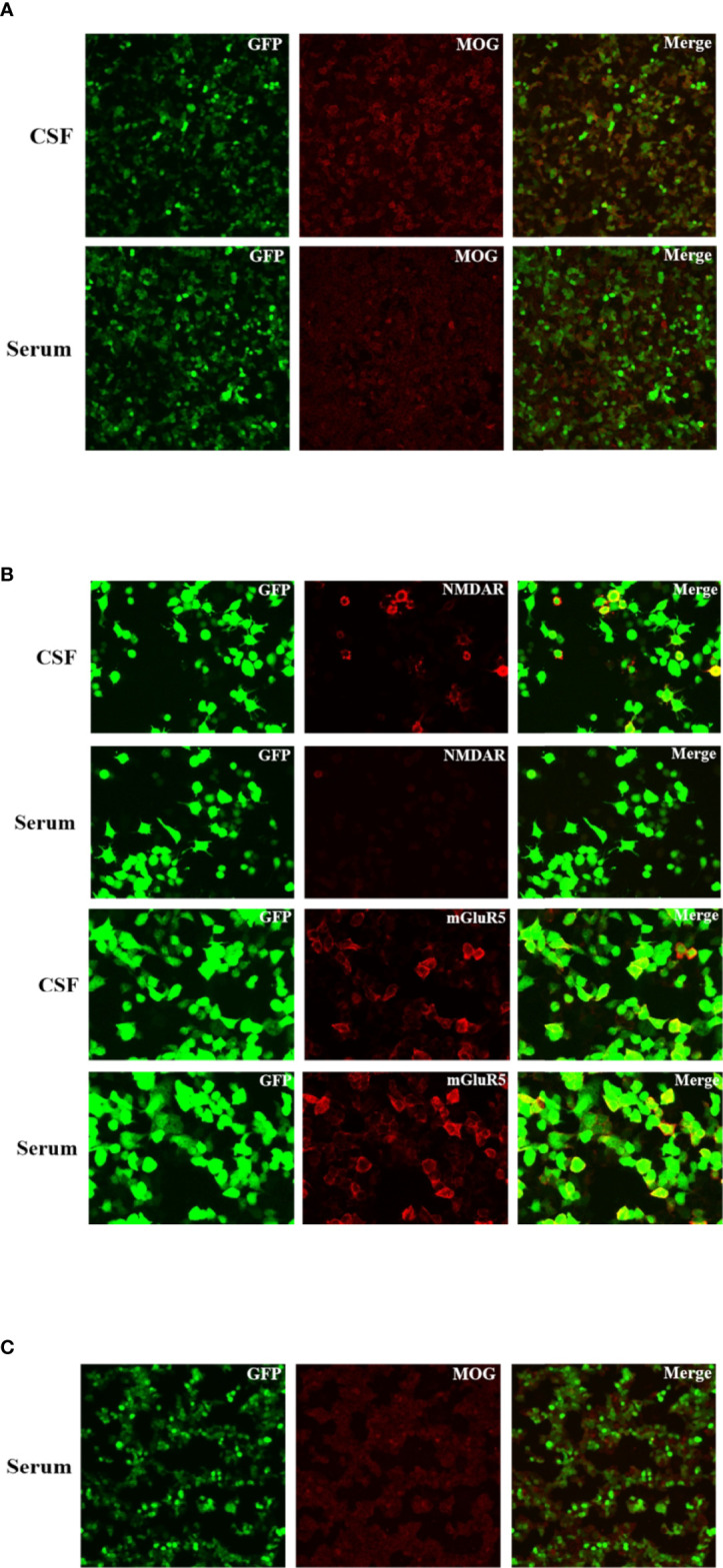
Immunofluorescence of anti-NMDAR, anti-mGluR5 and MOG antibodies in the patient’s cerebrospinal fluid and serum. These antibodies bound on the antigens expressed by the HEK293 cells and visualized by the immunofluorescence of fluorescein on the second antibody. **(A)** Fluorescent antibody staining for expression of MOG antibody in the serum and CSF of the patient at three weeks after the symptom onset. **(B)** Fluorescent antibody staining for expression of NMDAR and mGluR5 antibodies in the serum and CSF of the patient at three weeks after the symptom onset. **(C)** Fluorescent antibody staining for expression of MOG antibody in the serum of the patient at six months after discharge.

**Figure 3:**
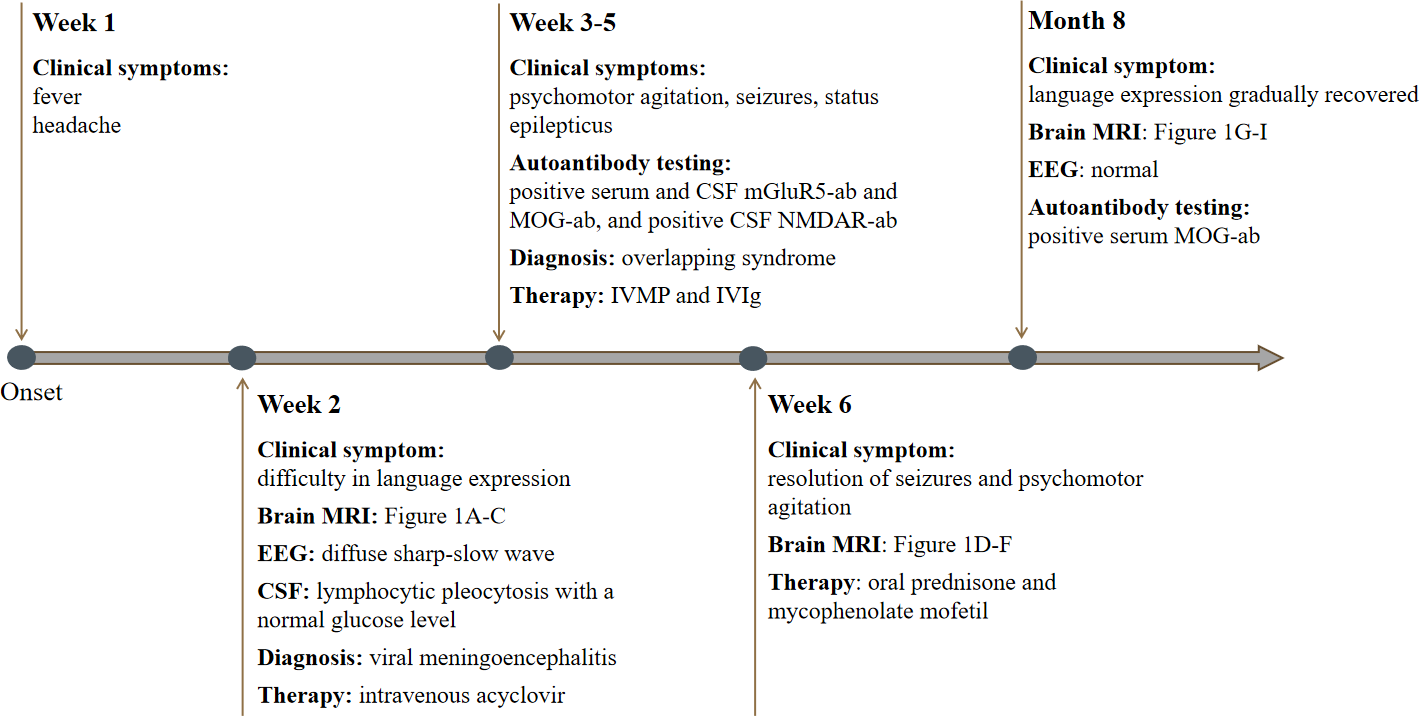
Timeline of our patient with clinical manifestations, relevant results of examinations, diagnosis and interventions.

On the seventh day of admission, the patient developed multiple episodes of psychomotor agitation, and he had an episode of generalized tonic-clonic seizure, all four limbs becoming rigid along with up-rolling of the eyes, which lasted for about two minutes and then resolved. The following day he developed convulsive status epilepticus, and intravenous diazepam 10 mg was administered to terminate the seizure activity. Subsequently, oral oxcarbazepine (300 mg b.i.d.) was given and antiviral treatment with intravenous acyclovir continued. Lumbar puncture was performed again, and the repeated CSF test still indicated leukocytosis (203 * 10^6^/L) and a high protein level (1.132 g/L). Meanwhile, CSF and serum testings for MOG antibody and autoimmune encephalitis panel (antibodies against IgLON5, DPPX, GlyR1, DRD2, mGluR5, NMDAR, AMPA1, AMPA2, LGI1, CASPR2, GABAB, mGluR1, GAD65, and Neurexin-3α) were conducted on a cell based assay (CBA). MOG antibodies were positive in the serum (1:32) and CSF (1:10) (**Figure 2A**). Autoimmune encephalitis panel revealed positivity for serum and CSF mGluR5 antibody (both 1:10) as well as positivity for CSF NMDAR antibody (1:10) (**Figure 2B**). He was diagnosed with an overlapping syndrome of MOG antibody-associated cortical encephalitis and NMDAR, mGluR5 antibody autoimmune encephalitis. He was treated with intravenous methylprednisolone (1000 mg/day for 3 days, 500 mg/day for 3 days, 250 mg/day for 3 days, and 120 mg/day for 3 days) and immunoglobulin (400 mg/kg/day for 5 days), leading to the resolution of seizures and psychomotor agitation. Repeat brain MRI indicated alleviated cortical swelling (**Figures 1D–F**). Subsequently, the patient was discharged, and he received a gradual tapering dose of oral prednisone (60 mg/day followed by tapering by 5 mg every two weeks) and mycophenolate mofetil (1000 mg/day). Follow-up continued for 6 months, and the patient recovered his language expression ability and no further relapse was recorded. The repeated MMSE was 30/30. The follow-up EEG was normal and brain MRI revealed no obvious abnormalities (**Figures 1G–I**). In addition, repeated CSF analysis revealed normal cell count and protein. Reexamination of MOG antibody and autoimmune encephalitis panel indicated negativity for serum and CSF mGluR5 and NMDAR antibodies as well as CSF MOG antibody, and that serum MOG antibody titer decreased to 1:10 (**Figure 2C**). The patient also underwent tumor screening including tumor marker screening, chest CT scan and abdominal ultrasound. No tumor has been detected so far. The clinical manifestations, important results of examinations, relevant diagnosis and interventions of the patient have been summarized in **Figure 3**.

## Discussion

In this study, we describe a rare case of an overlapping syndrome with the coexistence of MOG-IgG, NMDAR-IgG and mGluR5-IgG. MOG-IgG and NMDAR-IgG coexistence is possible, since their autoantigens coexpress on the surface of oligodendrocytes (7). The immune cells may attack the antigens of MOG and NMDAR when targeting oligodendrocytes, and subsequent generate corresponding antibodies in CSF and serum (8). Indeed, several cases have recently reported the co-occurrence of antibodies against both MOG and NMDAR (9–11). Hence, a new emerging concept referring to the coexistence of anti-MOG and anti-NMDAR antibodies is called MOGAD and anti-NMDAR encephalitis overlapping syndrome (MNOS) (11). Anti-mGluR5 encephalitis is very rare, and the coexistence of anti-mGluR5 encephalitis and MNOS has not yet been reported. It is elusive why MOG-IgG, NMDAR-IgG and mGluR5-IgG were present in our patient as their coexistence remains uncommon. Of note, mGluR5 was reported to play a role in regulating the differentiation of oligodendrocyte progenitor cells into oligodendrocytes (12), which indicated that mGluR5 may participate in myelin dysfunction. Therefore, we speculate that MNOS-related oligodendrocyte injury may be accompanied by changed mGluR5 signals, which might be a possible explanation of the co-occurrence of MOG-IgG, NMDAR-IgG and mGluR5-IgG in our patient. Low MOG-IgG titers in the current case have been noticed, and thus the possibility of false positive results should be with caution. Nevertheless, low positive MOG-IgG is reported to be meaningful in the correct clinical context such as in patients with encephalitis (13). Additionally, patients with acute attack of unilateral cortical encephalitis are considered to have a high pre-test probability for MOGAD and a low risk of false-positive MOG-IgG results (14). Hence, we surmise that the potential risk of false-positive results of MOG-IgG testing in our case may be relatively low. To our knowledge, this is the first reported case of coexistent MOG, NMDAR, mGluR5 antibodies in the same patient, which expands the clinical spectrum of the overlapping syndrome recently reported.

MOG antibody-related cerebral cortical encephalitis was first reported by Ogawa et al. (2), which is a rare clinical phenotype of MOGAD. The most prominent clinical manifestation of MOG cortical encephalitis is a seizure, which occurred in about 82.6% of patients (1). Other clinical features include fever, headache, and cerebral cortical symptoms such as aphasia, mental disorder, and memory impairment, etc (1). Brain MRI is characterized by grey matter lesions, particularly involvement of the cerebral cortex and sulcus, but not the subcortical and deep white matter (2, 15). In addition, increased intracranial pressure, CSF pleocytosis with elevated protein levels are common in MOG cortical encephalitis (16). Due to atypical clinical features and CSF changes, MOG cortical encephalitis may be misdiagnosed as infectious meningoencephalitis in the early stage, as seen in our case. In the present case, the patient was initially diagnosed with viral meningoencephalitis and received antiviral treatment. However, the progressive deterioration of this patient made us suspect the initial diagnosis, and further testing for autoantibodies was conducted. Finally, this patient was diagnosed as overlapping MOG antibody-associated cortical encephalitis and NMDAR, mGluR5 antibody autoimmune encephalitis according to characteristic clinical features, neuroimaging characteristics and autoantibodies test in CSF/serum.

Anti-NMDAR encephalitis is the most common type of autoimmune encephalitis. The main clinical manifestations include seizure, psychosis, speech disorder, dyskinesia, impaired consciousness, and autonomic dysfunction (17). Anti-NMDAR encephalitis has been reported in association with CNS demyelinating diseases such as acute disseminated encephalomyelitis (18) and myelitis (19). Recently, anti-NMDAR encephalitis with the coexistence of MOG-IgG has also been observed. In an analysis of 691 patients with anti-NMDAR encephalitis, 12 patients demonstrated positivity for MOG-IgG at the same time (20). Of note, despite seizures being the most prominent symptom in MOG cortical encephalitis, status epilepticus is an unusual presentation of this disease (21). Contrastly, status epilepticus is relatively common in patients with anti-NMDAR encephalitis, as 25% of patients have been reported to present with status epilepticus (22). In our case, the patient developed convulsive status epilepticus, which may be attributed to overlapping anti-NMDAR encephalitis. Therefore, we suggest that patients with MOG cortical encephalitis should be simultaneously tested for other autoantibodies in the CSF and serum for the early detection of an overlapping autoimmune syndrome.

Anti-mGluR5 encephalitis is rare, but case reports of this disease have been increasing with antibody testing becoming more widely available. The occurrence of mGluR5 autoantibody was initially found in two patients with Ophelia syndrome, which refers to neuropsychiatric abnormalities and coexisting Hodgkin lymphoma (23). Given that neuropsychiatric disorders are also main clinical features of MOG cortical encephalitis and anti-NMDAR encephalitis, psychomotor agitation our patient had could be mediated by one or more of the three antibodies, but the extent of contribution of each antibody to the occurrence of the symptom is uncertain. In addition, anti-mGluR5 encephalitis can occur with tumors other than Hodgkin lymphoma or without tumor. Spatola M et al. (24) reported eleven cases of encephalitis with mGluR5, and they found that five out of eleven patients had no tumor and one patient was associated with small cell lung cancer. Of note, this study observed that in all patients with Hodgkin lymphoma, neuropsychiatric symptoms preceded the tumor diagnosis by 2-11 months. Therefore, they proposed that detection of mGluR5 antibody may be useful to anticipate the diagnostic of a cancer (6). In the present case, the initial testing of mGluR5 antibody was positive in serum and CSF, while the repeated examination of serum and CSF mGluR5 antibody performed 6 months after the discharge came negative, and the follow-up workup for malignancy was negative. However, long-term follow up is still necessary due to the possibility of anti-mGluR5 encephalitis relapse (24).

Despite the coexistence of MOG, NMDAR and mGluR5 antibodies, the initial therapy remains similar to that of a patient with single antibody positive. Generally, patients with MOGAD, either alone or as part of an overlapping syndrome, seem to exhibit a rapid response to steroid therapy (3, 25). However, prognostic features of overlapping syndromes may differ from those of isolated antibody disease. It has been reported that patients with overlapping syndromes may have a higher risk of recurrence, and the response to first-line treatments may be more variable (9). Currently, overlapping syndromes reported in most of published cases are mainly dual autoantibodies positivity, while our patient was characterized by triple positivity (MOG-IgG, NMDAR-IgG and mGluR5-IgG), whether this type of encephalitis leads to a worse prognosis remains unclear. In our case, the follow-up testing for autoantibodies indicated that serum and CSF NMDAR and mGluR5 antibodies as well as CSF MOG antibody were negative while serum MOG antibody was positive. Given the persistent positivity to serum MOG-IgG in our patient, continued immunomodulatory therapy and the regular workup of antibody titer during follow-up are also necessary.

This case highlights the difficulties in diagnosing MOG cortical encephalitis overlapping with autoimmune encephalitis due to the atypical clinical features and CSF changes, which may be easily misdiagnosed as infectious meningoencephalitis in the early stages. In patients with fever, headache, epileptic seizures and unilateral cortical MRI encephalitis, the simultaneous determination of autoimmune encephalitis antibodies and MOG antibody should be carried out at the earliest, in order to avoid missed diagnosis as well as initiate immunotherapy in a timely manner. Since MOG cortical encephalitis may cause severe clinical consequences and even deaths, early diagnosis and treatment are of importance (1). Additionally, although MNOS is unlikely associated with tumors (11), regular tumor screening is also relevant and important for patients with MNOS overlapping with anti-mGluR5 encephalitis, given anti-mGluR5 encephalitis probably accompanied by malignancies.

## Concluding remarks

We report a rare case of an overlapping syndrome with the coexistence of MOG-IgG, NMDAR-IgG and mGluR5-IgG, which widens the clinical spectrum of the overlapping syndrome recently reported. In patients with unexplained encephalitis, CSF and serum testing that covers both autoimmune encephalitis and CNS demyelinating diseases autoantibodies including MOG-IgG should be considered, in order to make an accurate diagnosis as well as administer adequate and timely treatment.

## Patient perspective

When I had headache and fever at onset, I thought that I just caught a cold. However, when I could not express what I want fluently and suffered from seizures, I felt very frightened. As therapy went on, my symptoms gradually improved, and I began to gain confidence and hope. At present, my daily life returns to normal, but I’m still a little worried about relapse of the disease.

## Data availability statement

The original contributions presented in the study are included in the article/**Supplementary Material**. Further inquiries can be directed to the corresponding author.

## Ethics statement

This study was reviewed and approved by Clinical Trial Ethics Committee of Affiliated Hospital of Southwest Medical University. The patients/participants provided their written informed consent to participate in this study. Written informed consent was obtained from the individual(s) for the publication of any potentially identifiable images or data included in this article.

## Author contributions

All authors have contributed significantly to the manuscript and approved it for publication. JF and BR conceived the case report idea. JF, LP, YY and YX collected and analyzed the case materials. The manuscript was written by JF, and reviewed by ZL and BR. All authors contributed to the article and approved the submitted version.

## Funding

This study was supported by the Doctoral Research Initiation Fund of Affiliated Hospital of Southwest Medical University (No. 21024).

## Acknowledgments

We appreciate the technical assistance provided by the KingMed Diagnostic laboratory in Antibody test and Cell-Based Assay.

## Conflict of interest

The authors declare that the research was conducted in the absence of any commercial or financial relationships that could be construed as a potential conflict of interest.

## Publisher’s note

All claims expressed in this article are solely those of the authors and do not necessarily represent those of their affiliated organizations, or those of the publisher, the editors and the reviewers. Any product that may be evaluated in this article, or claim that may be made by its manufacturer, is not guaranteed or endorsed by the publisher.

## Supplementary material

Supplementary Figure 1 The patient’s electroencephalogram at admission showed diffuse sharp-slow wave activity in left frontal and temporal lobes.

## References

[1] Shu H Ding M Shang P Song J Lang Y Cui L . Myelin oligodendrocyte glycoprotein antibody associated cerebral cortical encephalitis: Case reports and review of literature. Front Hum Neurosci (2022) 15:782490. doi: 10.3389/fnhum.2021.782490 35046784 PMC8762331

[2] Ogawa R Nakashima I Takahashi T Kaneko K Akaishi T Takai Y . MOG antibody-positive, benign, unilateral, cerebral cortical encephalitis with epilepsy. Neurol Neuroimmunol Neuroinflamm (2017) 4(2):e322. doi: 10.1212/NXI.0000000000000322 28105459 PMC5241006

[3] Wang YF Liu XW Lin JM Liang JY Zhao XH Wang SJ . The clinical features of FLAIR-hyperintense lesions in anti-MOG antibody associated cerebral cortical encephalitis with seizures: Case reports and literature review. Front Immunol (2021) 12:582768. doi: 10.3389/fimmu.2021.582768 34177880 PMC8231650

[4] Fujimori J Takahashi T Kaneko K Atobe Y Nakashima I . Anti-NMDAR encephalitis may develop concurrently with anti-MOG antibody-associated bilateral medial frontal cerebral cortical encephalitis and relapse with elevated CSF IL-6 and CXCL13. Mult Scler Relat Disord (2021) 47:102611. doi: 10.1016/j.msard.2020.102611 33160141

[5] Káradóttir R Cavelier P Bergersen LH Attwell D . NMDA receptors are expressed in oligodendrocytes and activated in ischaemia. Nature (2005) 438:1162–6. doi: 10.1038/nature04302 PMC1416283 16372011

[6] Guevara C Farias G Silva-Rosas C Alarcon P Abudinen G Espinoza J . Encephalitis associated to metabotropic glutamate receptor 5 (mGluR5) antibodies in cerebrospinal fluid. Front Immunol (2018) 9:2568. doi: 10.3389/fimmu.2018.02568 30455705 PMC6230718

[7] Ren Y Chen X He Q Wang R Lu W . Co-Occurrence of anti-N-Methyl-D-Aspartate receptor encephalitis and anti-myelin oligodendrocyte glycoprotein inflammatory demyelinating diseases: A clinical phenomenon to be taken seriously. Front Neurol (2019) 10:1271. doi: 10.3389/fneur.2019.01271 31866928 PMC6904358

[8] Cherian A Divya KP Shetty SC Kannoth S Thomas B . NMDAR, CASPR2 antibody positivity: Triumph over the triumvirate. Mult Scler Relat Disord (2020) 46:102468. doi: 10.1016/j.msard.2020.102468 32906000

[9] Pérez CA Agyei P Gogia B Harrison R Samudralwar R . Overlapping autoimmune syndrome: A case of concomitant anti-NMDAR encephalitis and myelin oligodendrocyte glycoprotein (MOG) antibody disease. J Neuroimmunol (2020) 339:577124. doi: 10.1016/j.jneuroim.2019.577124 31837635

[10] Li S Wang M Li H Wang J Zhang Q Zhou D . Case report: Overlapping syndrome of anti-NMDAR encephalitis and MOG inflammatory demyelinating disease in a patient with human herpesviruses 7 infection. Front Immunol (2022) 13:799454. doi: 10.3389/fimmu.2022.799454 35529871 PMC9074690

[11] Guo J Bu Y Liu W . Case report: A case with MOGAD and anti-NMDAR encephalitis overlapping syndrome mimicing radiological characteristics of CLIPPERS. Front Immunol (2022) 13:832084. doi: 10.3389/fimmu.2022.832084 35493443 PMC9047684

[12] Luyt K Váradi A Durant CF Molnár E . Oligodendroglial metabotropic glutamate receptors are developmentally regulated and involved in the prevention of apoptosis. J Neurochem (2006) 99:641–56. doi: 10.1111/j.1471-4159.2006.04103.x 16836654

[13] Reindl M Schanda K Woodhall M Tea F Ramanathan S Sagen J . International multicenter examination of MOG antibody assays. Neurol Neuroimmunol Neuroinflamm (2020) 7(2):e674. doi: 10.1212/NXI.0000000000000674 32024795 PMC7051197

[14] Sechi E Buciuc M Pittock SJ Chen JJ Fryer JP Jenkins SM . Positive predictive value of myelin oligodendrocyte glycoprotein autoantibody testing. JAMA Neurol (2021) 78(6):741–6. doi: 10.1001/jamaneurol.2021.0912 PMC8077043 33900394

[15] Hamid SHM Whittam D Saviour M Alorainy A Mutch K Linaker S . Seizures and encephalitis in myelin oligodendrocyte glycoprotein IgG disease vs aquaporin 4 IgG disease. JAMA Neurol (2018) 75:65–71. doi: 10.1001/jamaneurol.2017.3196 29131884 PMC5833490

[16] Yao T Zeng Q Xie Y Bi F Zhang L Xiao B . Clinical analysis of adult MOG antibody-associated cortical encephalitis. Mult Scler Relat Disord (2022) 60:103727. doi: 10.1016/j.msard.2022.103727 35320766

[17] Son DK Cho SM Ryu HU Shin BS Kang HG . Anti-NMDAR encephalitis with bilateral basal ganglia MRI lesions at a distance of time: a case report. BMC Neurol (2022) 22:121. doi: 10.1186/s12883-022-02652-y 35346099 PMC8962229

[18] Lekoubou A Viaccoz A Didelot A Anastasi A Marignier R Ducray F . Anti-N-methyl-D-aspartate receptor encephalitis with acute disseminated encephalomyelitis-like MRI features. Eur J Neurol (2012) 19:e16–7. doi: 10.1111/j.1468-1331.2011.03617.x 22182357

[19] Pennington C Livingstone S Santosh C Razvi S . N-methyl d-aspartate receptor antibody encephalitis associated with myelitis. J Neurol Sci (2012) 317:151–3. doi: 10.1016/j.jns.2012.02.034 22459355

[20] Titulaer MJ Höftberger R Iizuka T Leypoldt F McCracken L Cellucci T . Overlapping demyelinating syndromes and anti-N-methyl-D-aspartate receptor encephalitis. Ann Neurol (2014) 75:411–28. doi: 10.1002/ana.24117 PMC4016175 24700511

[21] Cobo-Calvo Á Ruiz A D'Indy H Poulat AL Carneiro M Philippe N . MOG antibody-related disorders: common features and uncommon presentations. J Neurol (2017) 264:1945–55. doi: 10.1007/s00415-017-8583-z 28770374

[22] Liu Y Ma L Ma X Ma X Li J Li D . Simple and effective serum biomarkers potential for predicting status epilepticus in anti-N-methyl-D-aspartate receptor encephalitis. BMC Neurol (2022) 22:27. doi: 10.1186/s12883-021-02545-6 35031011 PMC8759236

[23] Carr I . The Ophelia syndrome: memory loss in hodgkin's disease. Lancet (1982) 1:844–5. doi: 10.1016/S0140-6736(82)91887-6 6122069

[24] Spatola M Sabater L Planagumà J Martínez-Hernandez E Armangué T Prüss H . Encephalitis with mGluR5 antibodies: Symptoms and antibody effects. Neurology (2018) 90:e1964–72. doi: 10.1212/WNL.0000000000005614 PMC5980520 29703767

[25] Sarigecili E Cobanogullari MD Komur M Okuyaz C . A rare concurrence: Antibodies against myelin oligodendrocyte glycoprotein and n-methyl-d-aspartate receptor in a child. Mult Scler Relat Disord (2019) 28:101–3. doi: 10.1016/j.msard.2018.12.017 30590238

