# Supplementary figures and images for: Case report: Overlapping syndrome mimicking infectious meningoencephalitis in a patient with coexistent MOG, NMDAR, mGluR5 antibody positivity
Source: Front Immunol. 2022 Aug 5;13:919125. doi: 10.3389/fimmu.2022.919125 (PMC9389075; doi:10.3389/fimmu.2022.919125)

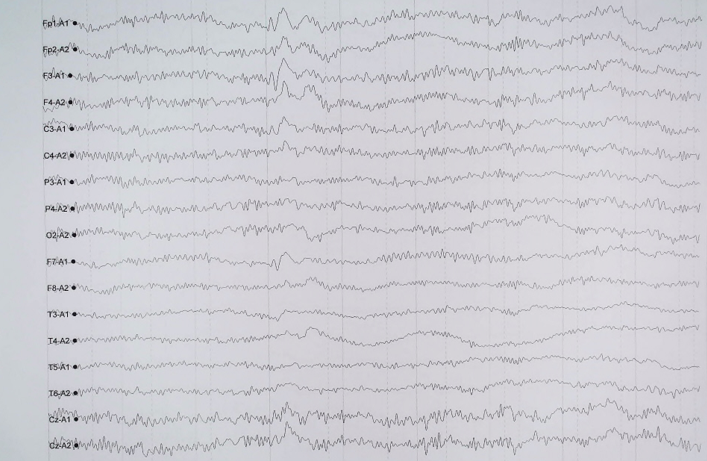

Supplement: Supplementary Figure 1 — The patient’s electroencephalogram at admission showed diffuse sharp-slow wave activity in left frontal and temporal lobes. [file Image_1.tif]
